# Physical and Performance Characteristics of 3×3 Professional Male Basketball Players

**DOI:** 10.3390/sports11010017

**Published:** 2023-01-12

**Authors:** Dimitrije Cabarkapa, Darko Krsman, Damjana V. Cabarkapa, Nicolas M. Philipp, Andrew C. Fry

**Affiliations:** 1 Jayhawk Athletic Performance Laboratory—Wu Tsai Human Performance Alliance, Department of Health, Sport and Exercise Sciences, University of Kansas, Lawrence, KS 66045, USA; 2 International Strength and Conditioning Institute, 21000 Novi Sad, Serbia

**Keywords:** sport, coaching, training, heart rate, agility, sprint, strength, vertical jump

## Abstract

Despite exponential growth in popularity over the last decade and recently becoming an Olympic sport, the amount of scientific literature focused on depicting a profile of successful 3×3 basketball players is sparse. Thus, the purpose of this study was to present the physical and performance characteristics of professional 3×3 male basketball players and how they differ between elite and non-elite athletes. The anthropometrics, vertical jump, agility, and sprint performance parameters collected from ten players during regular training sessions were ($\bar{x}$ ± SD): height (193.7 ± 4.5 cm), weight (89.2 ± 4.1 cm), wingspan (196.5 ± 5.2 cm), squat jump (43.5 ± 4.6 cm), countermovement jump with (53.3 ± 4.4 cm) and without an arm swing (46.3 ± 4.0 cm), reactive strength index (2.4 ± 0.3 m/s), *t*-test (10.3 ± 0.3 s), 505 drill (2.4 ± 0.2 s), 10 m sprint (1.5 ± 0.1 s), 30 m sprint (4.0 ± 0.3 s), shuttle run (27.7 ± 1.7 s), and bench press (98.2 ± 10.0 kg) and back squat (139.5 ± 17.6 kg) one repetition maximum. Additionally, the average and maximal heart rate (HR) responses during simulated games were 160.6 ± 8.0 and 188.5 ± 6.3 bpm, with players spending 6.3 ± 4.2, 11.4 ± 5.2, 13.9 ± 3.5, 26.4 ± 10.4, and 42.1 ± 10.0% of the total time in HR Zones 1–5, respectively. Interestingly, no statistically significant differences in the aforementioned physical and performance parameters were noted between elite and non-elite players. Overall, the findings of the present study provide coaches, sports scientists, and strength and conditioning practitioners with information that can aid in the athlete selection process, detection of areas for further improvement, and development of training regimens that resemble 3×3 basketball on-court competitive demands.

## 1. Introduction

Over the last decade, 3×3 basketball has experienced exponential growth in popularity worldwide. In 2020, 3×3 basketball has officially become an Olympic sport. The fast-paced style of play and rapid changes in score make this game appealing to a large spectrum of audiences. Although it has much in common with traditional 5×5 basketball, there are some fundamental differences in game rules and regulations between these two competitive styles. For example, smaller court size (11 × 15 m), single basket, fewer players (3 + 1 substitute), and a 12-s shot clock [1]. Additionally, it is important to note that despite being the same weight, the ball used in 3×3 basketball is smaller by 2.54 cm in diameter than the one used in a 5×5 basketball (i.e., 74.93 cm). Overall, the aforementioned differences may induce changes in on-court physiological and tactical demands [2,3].

Previous research has been primarily directed toward examining the physical and performance characteristics of 5×5 basketball players [4,5,6,7,8,9,10,11,12]. Considering the nature of the game of basketball, it is understandable why some of the most commonly researched topics pertain to player’s anthropometric attributes [7], vertical jump capabilities [5], maximal upper- and lower-body strength [6,13], and agility and sprint performance [4]. This information can allow coaches, sports scientists, and strength and conditioning practitioners to obtain a better insight into the profile of successful basketball players and adequately adjust off- and on-court training regimens [7,10,13].

On the other hand, the amount of scientific literature focused on examining the physical and physiological characteristics of 3×3 basketball players is sparse [1,2,3]. When examining performance profiles and game-related statistics across eight 3×3 basketball world cup games, Conte et al. [1] found that most live and stoppage time phases lasted <20 s, with the ratio between them being on average 0.92 ± 0.13. This is notably lower when compared to work-to-rest ratios observed within various 5×5 basketball competitive levels, which can be 1:2 or higher [14]. These findings are further supported by Leite et al. [3], indicating that the 3×3 competitive format elicited a notably greater rate of perceived exertion and maximal heart rate responses. In addition, a recently published study revealed that 3×3 basketball games were characterized by a greater number of ball touches, dribble drives, and long-distance shots when compared to the 5×5 competitive format [2].

Therefore, to bridge a gap in the scientific literature and provide sports practitioners with information that can be used to enhance on-court playing performance, the purpose of this study was to present the profile of 3×3 professional male basketball players and examine differences in physical and performance characteristics between elite and non-elite athletes.

## 2. Materials and Methods

### 2.1. Participants

Physical and performance characteristics examined in the present study were obtained from ten professional 3×3 male basketball players. All testing procedures were randomly conducted as a part of regular training regimens (i.e., 1–2 times per day, 4–6 per week) administered by their respective certified strength and conditioning specialists. The approval to gain access to and publish this data was acquired from the University’s Institutional Review Board.

### 2.2. Anthropometric Measurements

The athlete’s height, measured to the nearest centimeter, and weight, measured to the nearest tenth of a kilogram, were assessed by a portable stadiometer (SECA 213, Hamburg, Germany) and scale (BC-613, Tanita Corp., Tokyo, Japan), respectively. A measuring tape (H-300, Hoechstmass, Sulzbach, Germany) was used to measure the athlete’s wingspan (i.e., the distance between the tips of the middle fingers of each hand) to the nearest centimeter while holding arms away from the body parallel to the ground.

### 2.3. Vertical Jump Measurements

Upon completion of a standardized warm-up procedure consisting of dynamic stretching exercises (e.g., high-knees, A-skips, lunge-and-twist, and butt kicks), each athlete completed three maximum-effort squat jumps, drop jumps (i.e., 40 cm box height), countermovement vertical jumps without an arm swing (CVJ-NS), and countermovement vertical jumps with an arm swing (CMJ-S). To minimize the possible influence of fatigue, the aforementioned tests were spread across four training sessions and each jump trial was separated by a 30–60 s rest interval.

When performing squat jumps, drop jumps, and CMJ-NS, athletes were instructed to keep their hands on the hips during the entire movement. On the other hand, when performing CMJ-S, athletes were instructed to start with arms positioned slightly in the front of the body with elbows flexed at a 90-degree angle and finish with the arms extended above the head. All athletes were verbally encouraged to give maximal effort and focus on pushing away from the ground as explosively as possible [15]. The best score obtained from three total attempts for each jump test was recorded and used for performance analysis purposes.

The My Jump 2 application, downloaded on iPhone (Version 13, Apple Inc., Cupertino, CA, USA), was used to calculate jump height for a squat jump, CMJ-NS, and CMJ-S, and reactive strength index (RSI = flight time/ground contact time) for drop jump. Based on recently published research reports, this smartphone application has been demonstrated as a valid and reliable tool for the assessment of vertical jump performance when compared to a force plate and photocell system as criterion-measurement [16,17].

### 2.4. Agility and Sprinting Tests

To minimize the possible influence of fatigue, agility and sprinting tests were conducted separately. T-test and 505 drill were performed during the first training session, and 10 and 30 m sprints and shuttle run during the second training session. Prior to each training session, all athletes completed a standardized warm-up procedure consisting of dynamic stretching exercises (e.g., high-knees, A-skips, lunge-and-twist, and butt kicks), followed by two practice 30 m sprints at 50–70% of their estimated maximal intensity. Each athlete completed two trials separated by a 1–2-min rest interval, with a better score being recorded and used for performance analysis purposes. All athletes were verbally encouraged to give maximal effort.

**Figure 1:**
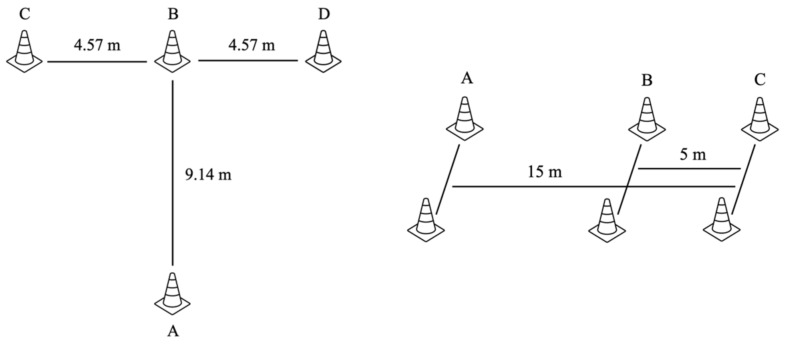
Graphical representation of the *t*-test and 505 drill.

For the *t*-test drill (Figure 1), the athlete started at cone A. On the coach’s command (i.e., 3-2-1-go), the athlete sprinted straight toward cone B and touched the tip of the cone with the right hand. The height of the cone was 30.48 cm. Then, the athlete shuffled sideways toward cone C, touched the tip of the cone with the left hand, and continued shuffling toward cone D. After touching cone D with the right hand, the athlete shuffled back to cone B, touching it with the left hand, and running backward toward cone A. The trial did not count if the athlete crossed the legs while shuffling and/or failed to touch the tip of each cone with the respective hand [4]. The stopwatch, measuring to the nearest tenth of a second, started on the “go” command and stopped when the athlete passed cone A.

Three sets of cones were used to mark 0, 10, and 15 m distances for the 505 drill (Figure 1). The athletes were instructed to stand in a split stand with their dominant foot behind the line positioned at cone A. On the coach’s command (i.e., 3-2-1-go), the athlete sprinted straight toward cone C, passing through a line marked by cone B. After touching the 15 m line with their dominant foot marked by cone C, the athletes changed direction and sprinted back as quickly as possible through the 10 m finish line marked by cone B. The stopwatch, measuring to the nearest tenth of a second, started on the “go” command and stopped when the athlete crossed a line between cone B.

**Figure 2:**
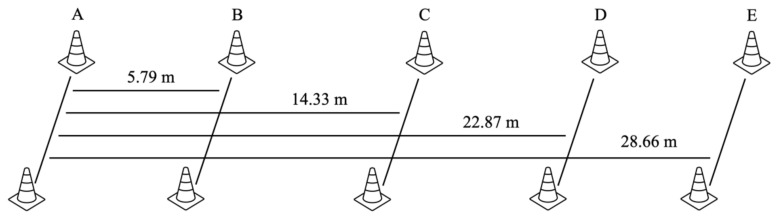
Graphical representation of shuttle run.

For 10 and 30 m sprints, the athletes were instructed to start in a split-stand position with their dominant foot positioned behind the line, and on the coach’s command (i.e., 3-2-1-go), run straight forward with maximal effort passing through a finish line. On the other hand, when performing a shuttle run (Figure 2), the athletes were instructed to start in the same position at cone A and change the direction of running multiple times. As their foot touched the line marked by cones B, C, D, and E, they sprinted back to the original position [18]. If the athlete failed to touch the line with the foot, the sprint trial did not count, and the run was repeated. The stopwatch, measuring to the nearest tenth of a second, started on the “go” command and stopped when the athlete crossed the finish line for 10 and 30 m sprints, and when the athlete passed cone A for the shuttle run. All athletes were verbally encouraged to give maximal effort through all testing procedures.

### 2.5. Strength Measurements

The one-repetition maximum (1RM) testing procedures for the barbell bench press resistance exercise followed the guidelines established by the National Strength and Conditioning Association [19]. Athletes were instructed to lie on the bench in a supine position (i.e., five-point body contact), grasp the barbell (Echo Bar 20 kg; Rogue, Columbus, OH, USA) with a closed pronated grip shoulder-width apart, and perform repetitions with the barbell positioned over the chest with maximal effort. Athletes performed the first set of 5–10 repetitions at self-selected light-to-moderate weight, followed by heavier sets of 3–5 repetitions. After each successfully completed lift, the weight was successively increased by 5–10%, until the maximum amount of weight that the athlete is capable of lifting was reached. Each set was separated by a two-minute rest interval to minimize the possible influence of fatigue [13].

The 1RM for back squat exercise was estimated based on the force-velocity profile for each athlete via an innovative PUSH smartphone application (Whoop Unite, Boston, MA, USA) downloaded on iPhone (Version 13, Apple Inc., Cupertino, CA, USA). This application demonstrated high validity and reliability when compared to the linear position transduced as a gold-standard testing modality [20]. The athletes were instructed to grasp the barbell with a closed pronated grip and place it on the upper trapezius maximus at the base of the neck, hold the chest up and out, tilt the head slightly up, take one to two steps backward, position the feet shoulder-width apart, and perform repetitions with maximal effort [13]. After performing 5–10 repetitions of back squats on a three-dimensional squat machine (Fettle Fitness, Jeffersonville, GA, USA) at self-selected light-to-moderate weights, athletes performed three repetitions at five assigned loads ranging between 25–85% of their estimated back squat 1RM. Each set was separated by a two-minute rest interval to assure adequate recovery. The estimated 1RM value was recorded and used for performance analysis purposes.

### 2.6. Heart Rate Measurements

As a part of their regular training regimens, all athletes participated in a simulated 3×3 basketball game while wearing heart rate monitors (H10; Polar Electro Oy, Kempele, Finland) placed directly on the skin just below the sternum (i.e., right across xiphoid process). This system, with a sampling rate of 1000 Hz, demonstrated excellent detection of RR intervals (i.e., the time elapsed between two consecutive R-waves) throughout a wide range of activities [21]. The basketball size and rules corresponded to 3×3 international regulation standards. To determine exercise intensity, maximal heart rate (HRmax) and average heart rate (HRavg) measurements were obtained during a game and used for performance analysis purposes. Also, the amount of total game time spent in each of the following heart rate zones, expressed as a percentage of the HRmax, was calculated: Zone 1 (50–59.9%), Zone 2 (60–69.9%), Zone 3 (70–79.9%), Zone 4 (80–89.9%), and Zone 5 (>90%).

### 2.7. Statistical Analysis

A Shapiro–Wilk’s test corroborated that all variables met the assumption of normality. Descriptive statistics, means, and standard deviations were calculated for each dependent variable. Independent *t*-tests were used to examine statistically significant differences in physical and performance characteristics between six elite (e.g., Masters and Challenger tournaments) and four non-elite (e.g., regional tournaments) 3×3 basketball players. Due to the small sample size (n < 20), Hedge’s *g* was used to calculate the measure of effect size (i.e., *g* = 0.2 is a small effect, *g* = 0.5 is a moderate effect, and *g* > 0.8 is a large effect). Statistical significance was set a priori to *p* < 0.05. All statistical analyses were completed with SPSS (Version 26.0; IBM Corp., Armonk, NY, USA).

## 3. Results

**Figure 3:**
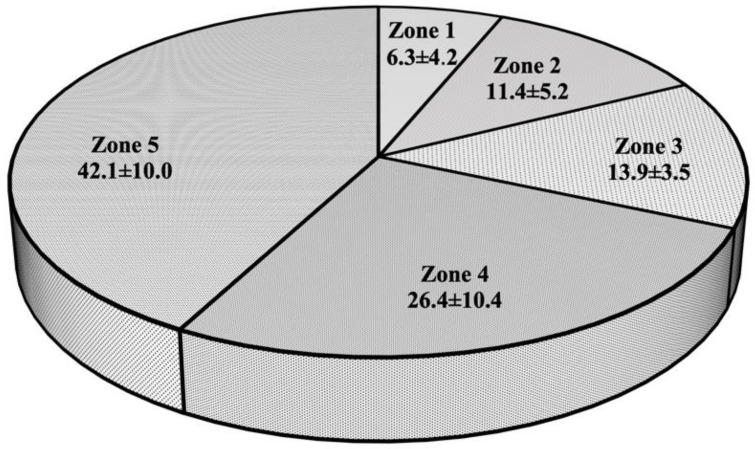
Graphical representation of heart rate zones [%] during 3×3 basketball game ($\bar{x}$ ± SD).

Dependent variables, means and standard deviations ($\bar{x}$ ± SD), for each physical and performance parameter examined in the present investigation are presented in Table 1 and Figure 3. No significant differences were observed in any variables between elite and non-elite 3×3 basketball players (all *p* > 0.05), except for age (*p* = 0.041), with elite players being slightly older. In addition, a small-to-moderate effect size was observed for CMJ-S, 30 m sprint, bench press 1RM, HRmax, and HR Zone 4, a moderate-to-large effect size for RSI, HR Zone 2 and 5, and a large effect size for HR Zone 1 pertaining to differences in physical and physiological characteristics between elite and non-elite players.

## 4. Discussion

The findings of the present study provide coaches, sports scientists, and strength and conditioning practitioners with detailed insight into physical and performance characteristics that can be used to develop a profile of 3×3 professional basketball players. This information may be of critical importance when identifying areas for individual player improvement and developing training regimens that adequately resemble on-court competitive demands.

The anthropometric characteristics (i.e., height, weight, and wingspan) obtained in the present investigation were similar to the findings of previous research reports focused on examining a broad spectrum of 5×5 basketball players, ranging from amateur to professional levels of competition [4,5,8,11,18,22]. The average height, weight, and wingspan measurements of NCAA Division-I basketball players reported collectively as a team in one of the recently published investigations were found to be 1.93 ± 0.08 m, 93.21 ± 15.09 kg, and 1.99 ± 0.10 m, respectively [22]. However, it is important to note that the magnitudes of the aforementioned physical characteristics may vary based on playing position in 5×5 basketball [10,23]. For example, the centers tend to have greater height, weight, and wingspan when compared to forwards and guards [10,23]. In 3×3 basketball, due to on-court playing demands, each player needs to know how to rebound, pass, shoot, and dribble the ball. Thus, the skill set of the 3×3 players tends to be more uniform, as there are no specifically assigned playing positions such as in 5×5 basketball. Moreover, the findings of the present study revealed no significant differences in anthropometric characteristics between elite and non-elite 3×3 basketball players. Although further research is warranted on this topic, we can assume that a player’s proficiency in performing basketball-specific tasks has a greater influence on determining success in 3×3 basketball than physical attributes such as height, weight, and wingspan.

A considerable amount of the scientific literature has been focused on examining the vertical jump characteristics of 5×5 basketball players, as one of the key parameters strongly related to on-court playing performance [4,5,10,12,18,24]. Squat jump, CMJ-NS, and CMJ-S have been widely used as testing modalities that sports practitioners implement to non-invasively evaluate athletes’ lower-body strength and power-producing capabilities [25]. Previous research has found the squat jump and CMJ-NS performance of elite junior 5×5 basketball players to be 39.8 ± 3.7 and 40.1 ± 4.0 cm, respectively [5]. When studying a large cohort of top-level 5×5 professional basketball players, Ostojic et al. [10] found that the CMJ-NS performance ranged between 31.3–89.9 cm, with an average value of 57.4 ± 7.7 cm. The discrepancy between the previously-mentioned findings and the results obtained in the present investigation may be related to the differences in competitive levels (e.g., junior vs. professional) as well as testing methodologies used for the assessment of vertical jump performance (e.g., force plate vs. smartphone application). On the other hand, jump heights attained during the CMJ-S were similar to the findings of Delextrat & Cohen [18] who focused on examining elite and average-level collegiate basketball players. The RSI of 3×3 basketball players was slightly higher than the values obtained for professional athletes when performing drop jumps from the same height (i.e., 40 cm box height) [26]. In addition, it is important to note that, despite not reaching the level of statistical significance, elite 3×3 basketball players tended to display slightly better RSI (moderate-to-large effect size) and CMJ-S (small-to-moderate effect size) performance when compared to non-elite athletes.

Considering that basketball is a sport that requires repetitive sprinting and change-of-direction movements, it is understandable that these performance attributes have been extensively studied in the scientific literature [4,18,27]. Previous research has found that elite level 5×5 professional basketball players’ performance on the *t*-test, 10 m, and 30 m sprint test can range between 9.3–10.4, 1.6–1.8, and 4.0–4.3 s, with mean values of 9.7 ± 0.3, 1.7 ± 0.1, 4.2 ± 0.2 s, respectively [27]. Interestingly, Alemdaroglu et al. [4] reported a faster *t*-test (9.3 ± 0.5 s) and slower 10 m (1.9 ± 0.3 s) and 30 m (4.3 ± 0.2 s) sprint times when examining a cohort of slightly older 5×5 professional basketball players. When compared to the results obtained in the present investigation, it is interesting to note that 3×3 basketball players demonstrated somewhat poorer agility and better sprinting performance than 5×5 players examined in the aforementioned studies [4,27]. This may be attributed to the differences in testing methodologies (e.g., timing gates vs. stopwatch) and on-court playing demands between these two competitive styles as 3×3 basketball players are required to continuously perform high-speed inertial movements that ultimately result in a greater number of accelerations, decelerations, and distance covered at high-intensity thresholds [2,28]. In addition, the times to complete the shuttle run (i.e., formerly known as the suicide run) were faster for 3×3 basketball players examined in the present study when compared to collegiate elite and average-level 5×5 basketball players [18]. However, the difference in the testing methodologies must be acknowledged, as the players in the present study did not dribble the ball while running the whole distance. Moreover, it should be noted that elite and non-elite 3×3 basketball players performed similarly on all agility and sprint performance tests. Although further research involving a larger sample size is necessary, these findings suggest that 3×3 basketball players need to be proficient in performing these types of movements, regardless of the competitive level.

Alongside agility and sprinting performance, the impotence of upper- and lower-body strength for optimal basketball performance has been well documented [6,13,24,29]. Previous research has found a strong positive relationship between the back squat (r = 0.52–0.64) and bench press 1RM (r = 0.71) and playing time at NCAA Division-I and II levels of basketball competitions [24,29]. Moreover, in a recently published study, Cabarkapa et al. [6] found a positive association between lower-body strength and power and post-collegiate playing opportunities, with greater values being associated with a greater level of play. The observed values for a cohort of 3×3 basketball players observed in the present investigation for back squat and bench press 1RM fall within the previously reported ranges for NCAA Division-I collegiate players, 54.5–186.4 and 81.8–262.3 kg, with mean values of 102.7 ± 19.9 and 152.2 ± 36.5 kg, respectively (Latin et al., 1994). On the other hand, the back squat and bench press 1RM in the present study were notably greater than the values reported by Cabarkapa et al. [13] for recreationally active individuals with previous basketball playing experience, which is expected considering the difference in the training status (e.g., professional vs. recreational). In addition, the back squat and bench press 1RM magnitudes were similar between elite and non-elite 3×3 basketball players, implying that both groups already possessed the required levels of upper- and lower-body strength. However, future research should focus on examining whether additional strength gains elicit further advancements in on-court playing performance.

Lastly, the findings of the present study offer further insight into HR responses that 3×3 basketball players experience during a simulated game. This information can be of critical importance for the development of appropriate training regimens that adequately resemble on-court playing demands [30]. Although influenced by playing position (e.g., center, guard), HRavg and HRmax during simulated 5×5 scrimmages and games were reported to be 147 ± 10 and 162 ± 7 bpm, and 171 ± 12 and 173 ± 6 bpm, respectively [8,31]. Interestingly, both elite and non-elite 3×3 basketball players examined in the present study demonstrated notably higher HRavg and HRmax values. Similar findings pertaining to differences in HR responses between 3×3 and 5×5 competitive formats were observed by Leite et al. [3] when studying youth basketball players. Additionally, the same authors have observed that 3×3 players spent a significantly greater amount of time in high-intensity zones (e.g., Zone 5, >90% HRmax) than 5×5 basketball players [3]. When taking into account the 3×3 rules and regulations, this is expected as fewer players on the court need to cover greater distances. Comparable observations were made by Delextrat & Kraiem [32], as players spent more time in low-to-moderate HR zones during 3×3 than in 2×2 basketball practice drills. While further research is warranted on this topic, due to the small sample size, it is interesting to note that elite 3×3 basketball players tend to spend less time in Zone 4 and 5, and more time in Zone 1 and 2 during a simulated game. Thus, these findings may imply that elite 3×3 players have more experience and better control over the game (e.g., tactical discipline), possibly due to being older. By knowing when high-intensity efforts are needed to adequately respond to the opponent’s offensive and defensive strategies, elite 3×3 basketball players may be able to optimize their recovery by spending more time in low-to-moderate HR zones (e.g., HR Zone 2).

In conclusion, the findings of the present study allow coaches, sports scientists, and strength and conditioning practitioners to develop a better profile of successful 3×3 basketball players. Obtaining a deeper insight into adequate levels of physical and performance parameters may help in the athlete selection process, detecting areas for further improvement, and developing training regimens that resemble 3×3 basketball on-court competitive demands.

## Figures and Tables

**Table 1:** Physical and performance characteristics of all 3×3 basketball players, and differences between elite and non-elite athletes ($\bar{x}$ ± SD).

Variable	All Players	Non-Elite	Elite	*p*-Value	Effect Size
Height [cm]	193.7 ± 4.5	193.3 ± 3.2	194.0 ± 5.5	0.814	0.147
Weight [kg]	89.2 ± 4.1	89.3 ± 5.7	89.2 ± 3.3	0.977	0.023
Wingspan [cm]	196.5 ± 5.2	196.8 ± 2.5	196.3 ± 6.7	0.910	0.091
Age [years] *	23.9 ± 4.1	21.0 ± 1.2	25.8 ± 4.3	0.041	1.380
Squat jump [cm]	43.5 ± 4.6	42.8 ± 5.7	44.0 ± 4.3	0.715	0.246
CMJ-NS [cm]	46.3 ± 4.0	46.6 ± 4.6	46.1 ± 4.0	0.864	0.118
CMJ-S [cm]	53.3 ± 4.4	52.5 ± 4.8	53.9 ± 4.4	0.665	0.307
RSI [m/s]	2.4 ± 0.3	2.3 ± 0.2	2.5 ± 0.3	0.180	0.749
*t*-test [s]	10.3 ± 0.3	10.3 ± 0.3	10.4 ± 0.4	0.581	0.293
505 drill [s]	2.4 ± 0.2	2.4 ± 0.1	2.4 ± 0.2	0.840	0.000
10 m sprint [s]	1.5 0.1	1.5 ± 0.1	1.5 ± 0.1	0.982	0.000
30 m sprint [s]	4.0 ± 0.3	4.0 ± 0.2	3.9 ± 0.4	0.259	0.342
Shuttle run [s]	27.7 ± 1.7	27.6 ± 1.5	27.7 ± 1.9	0.961	0.057
1RM bench press [kg]	98.2 ± 10.0	96.3 ± 4.8	99.5 ± 12.8	0.645	0.304
1RM back squat [kg]	139.5 ± 17.6	140.0 ± 21.2	139.2 ± 16.9	0.946	0.043
HRmax [bpm]	188.5 ± 6.3	187.3 ± 8.5	189. ± 5.1	0.638	0.304
HRavg [bpm]	160.6 ± 8.0	160.8 ± 10.3	160.5 ± 7.3	0.965	0.035
Zone 1 [%]	6.3 ± 4.2	2.9 ± 2.5	8.5 ± 6.9	0.176	0.988
Zone 2 [%]	11.4 ± 5.2	9.2 ± 3.5	12.8 ± 5.9	0.308	0.701
Zone 3 [%]	13.9 ± 3.5	14.0 ± 2.4	13.8 ± 4.3	0.947	0.054
Zone 4 [%]	26.4 ± 10.4	28.6 ± 15.6	24.9 ± 6.6	0.620	0.340
Zone 5 [%]	42.1 ± 10.0	45.4 ± 12.8	39.9 ± 8.2	0.435	0.541

CMJ-S—countermovement vertical jump with an arm swing; CMJ-NS—countermovement vertical jump without an arm swing; RSI—reactive strength index; 1RM—one repetition maximum; HRmax—maximal heart rate; HRavg—average heart rate; * significantly different when compared to non-elite (*p* < 0.05).

## Data Availability

The data presented in this study are available on request from the corresponding author.
